# A Systematic Framework for Molecular Dynamics Simulations of Protein Post-Translational Modifications

**DOI:** 10.1371/journal.pcbi.1003154

**Published:** 2013-07-18

**Authors:** Drazen Petrov, Christian Margreitter, Melanie Grandits, Chris Oostenbrink, Bojan Zagrovic

**Affiliations:** 1 Max F. Perutz Laboratories, University of Vienna, Campus Vienna Biocenter, Vienna, Austria; 2 University of Natural Resources and Life Sciences, Vienna, Austria; Fudan University, China

## Abstract

By directly affecting structure, dynamics and interaction networks of their targets, post-translational modifications (PTMs) of proteins play a key role in different cellular processes ranging from enzymatic activation to regulation of signal transduction to cell-cycle control. Despite the great importance of understanding how PTMs affect proteins at the atomistic level, a systematic framework for treating post-translationally modified amino acids by molecular dynamics (MD) simulations, a premier high-resolution computational biology tool, has never been developed. Here, we report and validate force field parameters (GROMOS 45a3 and 54a7) required to run and analyze MD simulations of more than 250 different types of enzymatic and non-enzymatic PTMs. The newly developed GROMOS 54a7 parameters in particular exhibit near chemical accuracy in matching experimentally measured hydration free energies (RMSE = 4.2 kJ/mol over the validation set). Using this tool, we quantitatively show that the majority of PTMs greatly alter the hydrophobicity and other physico-chemical properties of target amino acids, with the extent of change in many cases being comparable to the complete range spanned by native amino acids.

## Author Summary

Post-translational modifications, i.e. chemical changes of protein amino acids, play a key role in different cellular processes, ranging from enzymatic activation to transcription and translation regulation to disease development and aging. However, our understanding of their effects on protein structure, dynamics and interaction networks at the atomistic level is still largely incomplete. In particular, molecular dynamics simulations, despite their power to provide a high-resolution insight into biomolecular function and underlying mechanisms, have been limited to unmodified, native proteins due to a surprising deficiency of suitable tools and systematically developed parameters for treating modified proteins. To fill this gap, we develop and validate force field parameters, an essential part of the molecular dynamics method, for more than 250 different types of enzymatic and non-enzymatic post-translational modifications. Additionally, using this tool, we quantitatively show that microscopic properties of target amino acids, such as hydrophobicity, are greatly affected by the majority of modifications. The parameters presented in this study greatly expand the range of applicability of computational methods, and in particular molecular dynamics simulations, to a large set of new systems with utmost biological and biomedical importance.

## Introduction

Proteins in the cell continually get covalently modified in different post-translational, enzyme-controlled reactions [1]–[3]. Additionally, protein modifications frequently arise in a non-controlled fashion as well, mainly as a consequence of oxidative stress [4]. While enzymatic post-translational modifications (PTMs) play important regulatory roles in a large number of different cellular processes, non-enzymatic PTMs are predominantly linked with protein damage and are involved in age-related diseases such as neurodegenerative disorders, diabetes and cancer [2], [4]–[7]. Despite the general importance of PTMs in different biological contexts, their effect on protein structure, dynamics and interaction networks at the atomistic level remains poorly understood. In particular, molecular dynamics (MD) simulations, a widely used high-resolution computational method for studying biomolecular properties and behavior [8]–[10], have been limited to unmodified, native proteins due to a surprising deficiency of suitable tools and systematically developed parameters for treating PTMs, with only sporadic exceptions [11]–[16].

MD simulations capture atomic and molecular motions based on Newton's equation of motion and an empirical potential energy function that defines interactions between simulated particles. The latter is defined by a force field, i.e. a self-consistent set of physically realistic equations and semi-empirical parameters describing all interactions in a given system. Force-field parameters are typically obtained by fitting atomic or molecular properties of small molecules against calculated quantum-mechanical or experimentally measured data. As the applied parameterization strategies often differ from each other, considerably different parameter values have been derived in many cases [17]–[20]. Here, we develop force field parameters for over 250 different types of enzymatic and non-enzymatic modifications of amino-acid side chains as well as protein termini within the context of GROMOS 45a3 [19] and 54a7 [21], [22] force fields (Table S1). We choose GROMOS force fields because of their widespread usage, high accuracy in reproducing experimental results and general transferability of parameters when it comes to identical chemical groups in different compounds [21] (e.g. from the hydroxyl group of tyrosine to the hydroxyl group of 7-hydroxytryptophan). The functional form of a typical force field is exemplified in equation 1 for GROMOS class force fields,
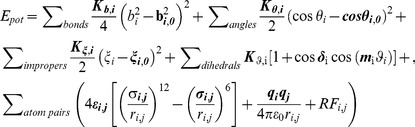
(1)with parameters highlighted using boldface letters and RF representing a reaction field contribution to the electrostatic interactions. The non-bonded interaction terms in the GROMOS force field are primarily parameterized against thermodynamic data of small molecules, either in the pure liquid state, or in aqueous or nonpolar solution. Therefore, we validate the obtained parameters by reproducing experimental hydration free energies (HFEs), a measure of hydrophobicity and arguably one of the most important amino-acid properties with implications in protein folding, ligand binding or protein-lipid interactions. Finally, we analyze physico-chemical properties related to hydrophobicity of all parameterized PTMs according to their type and compare them against the 20 canonical amino acids.

## Results

### Parameterization of PTMs

**Figure 1:**
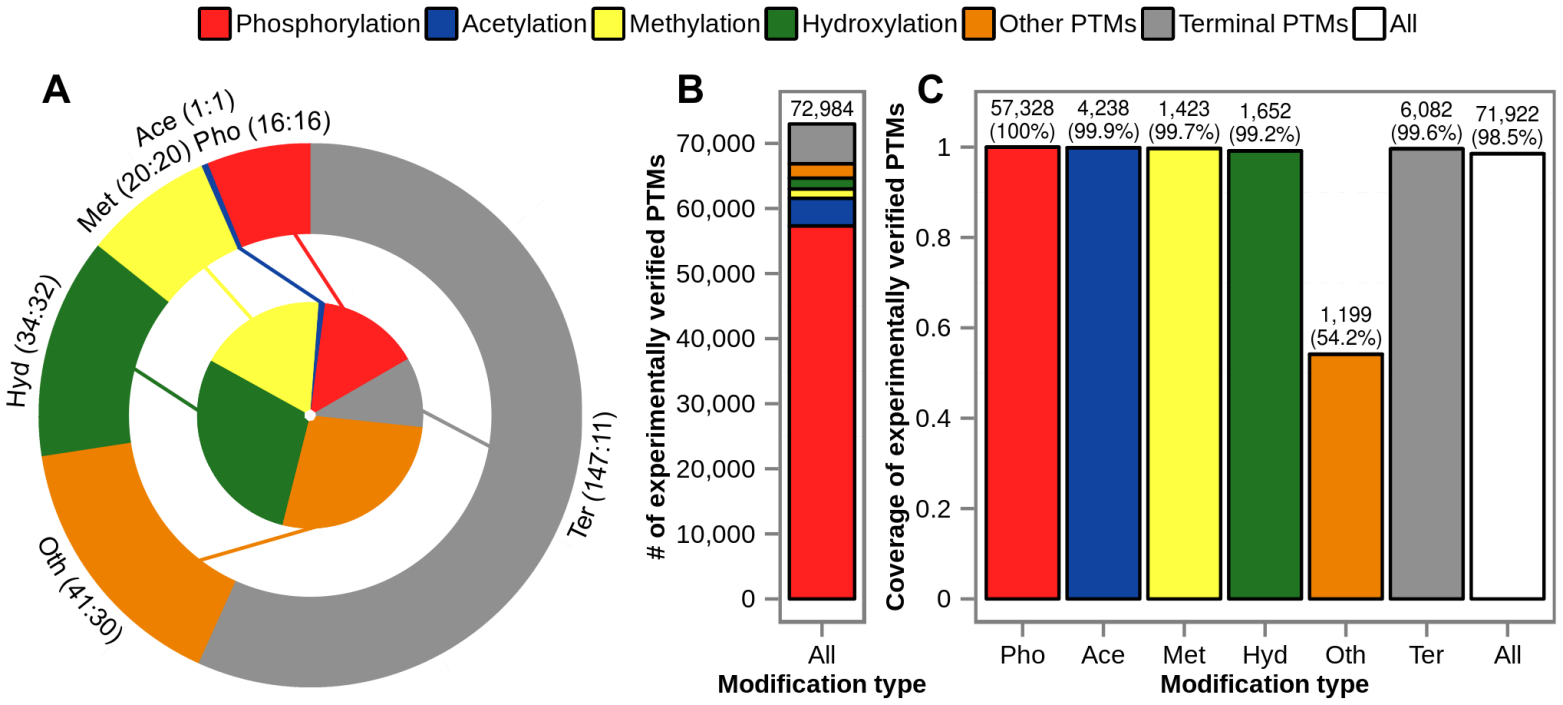
Summary of the number and coverage of parameterized PTMs. a) the number of parameterized PTMs by type (outer annulus) together with the number of parameterized non-redundant compounds by type (inner circle), labeled accordingly (number of PTMs: number of compounds); b) the number of experimentally verified PTMs by type annotated in the UniProt database (total of 72,984); c) coverage of experimentally verified PTMs shown as percentages with the values and the number of covered modifications displayed (top of bars). Color code: phosphorylation-red, acetylation-blue, methylation-yellow, hydroxylation-green, other PTMs-orange, terminal PTMs-gray and all-white.

One of the principal objectives in our parameterization has been the coverage of experimentally known PTMs, which is as complete as possible. Following an exhaustive literature search and analysis of an online PTM database PTMdb [23], we have compiled a diverse list of enzymatic and non-enzymatic PTMs, including phosphorylation, methylation, acetylation, hydroxylation, carboxylation, carbonylation, nitration, deamidation and many others (Figure 1a, Table S1), covering a total of 259 distinct PTM reactions or 110 non-redundant post-translationally modified amino acids and protein termini. The lower number in the latter case reflects the fact that different PTM reactions can lead to the same modified product (e.g. glutamic semialdehyde is a product of both arginine and proline carbonylation). We have generated GROMOS 45a3 (Dataset S1) and 54a7 (Dataset S2) force field parameters for the non-redundant set of compounds by either direct transfer or analogy to already parameterized compounds including amino acids, nitrogenous bases and other small molecules or completely novel parameterization (see Methods for more details).

How well do the obtained parameters cover the space of biologically relevant PTMs? To address this question, we have analyzed PTMs that have been experimentally verified (72,984) and annotated as such in the UniProt database [24] (21,411 protein entries, Dataset S3. Phosphorylation is by-far the most abundant modification type in the UniProt database (78.5% of all UniProt PTMs), followed by acetylation, hydroxylation and methylation (Figure 1b). Note that terminal PTMs account for a sizable fraction of all annotated modification at 8.3%. Strikingly, the parameterized compounds reported herein match every annotated phosphorylation modification, 99.9% of acetylation, 99.2% of hydroxylation and 99.7% of methylation modifications, for a grand-total coverage of 98.5% of all PTMs reported in UniProt (Figure 1c). Concerning PTMs that are not covered by our parameters, they are all extremely rare, each accounting for less than 0.5% of all UniProt PTMs. Finally, we provide parameters for 33 PTMs (Table S1), mostly non-enzymatic ones, that have to date not been reported in UniProt.

### Validation against experimental HFEs

HFE, a free energy difference between a compound solvated in water and the same compound in the gas phase, is an experimentally measurable property related to hydrophobicity, and it has been originally used to re-parameterize the GROMOS force field in 2004 [21]. A proper description of functional groups in the hydrated phase is of crucial importance for virtually all relevant biomolecular processes, so we have used the same thermodynamic quantity to validate the parameters obtained in the present study. To the best of our knowledge, experimental HFEs are available for the exact side chain analogs of 13 parameterized PTMs only and we have therefore in the validation set also included compounds, which are chemically related to PTM side chains for which no experimental HFEs were available, for a total of 26 different molecules (only a single representative compound was included for each group of PTMs involving the same chemical moiety, Table 1). Note that the additional compounds related to PTM side chains have been parameterized in the same way as the relevant PTMs.

**Table 1:** HFEs of the molecules in the validation set: comparison between experimental and calculated values using the GROMOS 54a7 parameter set.

Compound	HFE (kJ/mol)	
	experimental	ffG54a7
**Validation set. PTM-side-chain analogs**		
N-butylacetamide	−39.0	−37.9
*o*-cresol	−24.6	−24.8
*m*-cresol	−23.0	−28.2
2-methyl-2-propanol	−18.7	−15.1
2-methyl-1-propanol	−18.8	−18.6
propan-2-ol	−19.8	−16.1
N-methylacetamine	−41.9	−38.0
methylpropanoate	−12.3	−5.8
methylacetate	−13.1	−8.7
dimethylsulfide	−6.7	−9.1
butanal	−13.3	−10.8
propanal	−14.4	−11.8
butane	8.7	9.5
**Validation set. Compounds similar to PTM-side-chain analogs**		
diethylamine	−17.0	−11.7
trimethylamine	−13.4	−4.7
ethene	5.4	13.8
bromobenzene	−6.1	−8.6
aniline	−23.0	−25.8
acetophenone	−19.2	−16.3
N-methylformamide	−41.9	−39.5
chlorophenol	−19.0	−22.8
*2-nitrophenol	−19.2	−33.8
nitrobenzene	−17.2	−15.6
acetone	−16.1	−8.5
dimethylsulfoxide	−42.3	−39.4
methylsulfonylmethane	−42.2	−41.9
**RMSE**	**-**	**4.2**
**Additional compounds**		
#2-nitrophenol	−19.2	−17.4
3-nitrophenol	−40.3	−43.0
4-nitrophenol	−44.5	−44.2
4-methylimidazole (Nδ-H)	−42.9	−46.7
4-methylimidazole (Nε-H)		−62.0
1-methylimidazole (Nδ-H)	−35.2	−25.2
1-methylimidazole (Nε-H)		−34.5

The outlier 2-nitrophenol described by:

* parameters used for other nitro-containing compounds, and

# parameters derived to match the experimental HFE.

Experimental HFEs are taken from refs. [21], [48]–[50].

**Figure 2:**
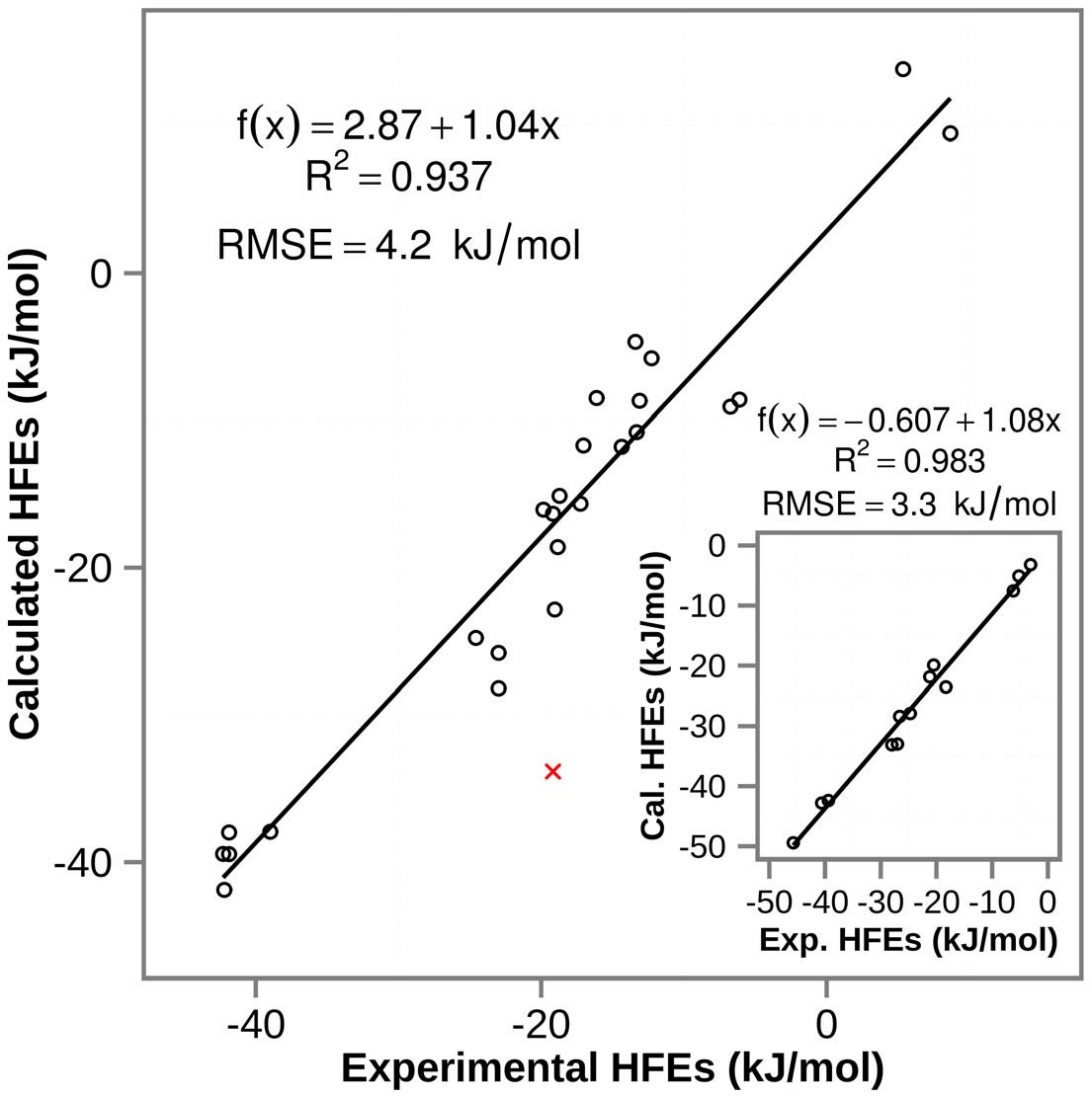
Experimental vs. calculated HFEs of compounds from the validation set (GROMOS 54a7). Correlation is captured by the regression line, its parameters, Pearson correlation coefficient and overall RMSE, with the outlier 2-nitrophenol in red (X symbol). The same comparison for canonical amino acids is shown in the inset. Note that error bars of calculated HFEs are comparable to the size of the symbols, with the average standard error of 0.4 kJ/mol.

We have used MD simulations and the thermodynamic integration (TI) approach [25] (see *Methods* for more details) to calculate the HFEs for neutral forms small-molecule analogs of the canonical amino-acid side chains and for the compounds in the validation set using both the 45a3 (Table S2) and 54a7 (Table 1) parameter sets of the GROMOS force field. As a consequence of the parameterization strategy behind them, the canonical amino acids exhibit an excellent agreement with experimental HFEs when it comes to the 54a7 parameter set, with a root-mean-square error (RMSE) of 3.3 kJ/mol (RT = 2.5 kJ/mol at room temperature) and an almost perfect correlation with experimental HFEs (correlation coefficient R^2^ = 0.98) (Figure 2). Remarkably, the newly generated GROMOS 54a7 force field parameters of PTM-related compounds exhibit a nearly equal level of matching of experimental HFEs with an RMSE of 4.2 kJ/mol (Table 1) and a correlation coefficient R^2^ of 0.94 (Figure 2) over 25 different compounds, excluding a single outlier, 2-nitrophenol (Figure 2, red X symbol). This compound, containing nitro and hydroxyl groups attached to a benzene ring, deviates from the experimental value by 14.6 kJ/mol.

Considering the outlier 2-nitrophenol in more detail, additional calculations have shown that *p*-cresol (a tyrosine side-chain analog), *o*-cresol, *m*-cresol and nitrobenzene, compounds containing either a hydroxyl group or a nitro group attached to a benzene ring, agree well with experimental HFEs with an overall RMSE of 2.7 kJ/mol only. This suggests that, although parameters of individual groups do reproduce experimental HFEs, the agreement with experiment may significantly worsen if they appear in combination. In order to test this, we have calculated HFEs of 3- and 4-nitrophenol and compared them against experimental values. Interestingly, the calculated HFEs of both compounds match experimental values (Table 1) suggesting either that these groups exert a specific influence on each other only in 2-nitrophenol or that the experimentally measured HFE may simply not be reliable for this compound. To account for the former possibility, we have derived a set of parameters *de novo* for 2-nitrophenol that closely match its experimental HFE with an absolute value of the deviation of 1.8 kJ/mol (Table 1). Note that we report both versions of nitrotyrosine (Table S1), a cognate PTM to 2-nitrophenol.

Finally, we have also excluded 4-methylimidazole (a histidine side-chain analog) and 1-methylimidazole from the HFE analysis of the canonical amino acids and PTMs, respectively, even though experimental HFEs are available for both compounds. Since histidine exists in two tautomeric states, described by different parameters, the calculated HFE depends on the choice of the state used for calculations, with one matching the experimental HFE and the other varying by approximately 20 kJ/mol (Table 1). Consequently, the same problem exists for 1′- and 3′-methylhistidine, whose parameters are based on those of histidine, where one tautomer matches while the other deviates from the experimental HFE (Table 1).

In contrast to GROMOS 54a7, the 45a3 parameter set does not reproduce experimental HFEs well (Table S2 and Figure S1). Namely, the slope of 0.79 and the offset of 3.8 kJ/mol of the regression line suggest that the calculated HFEs are largely overestimated (RMSE = 10.8 kJ/mol) for the amino-acid side chain analogs, as observed previously [21]. The same effect persists for the PTM compounds, with a RMSE from experimental HFEs of 15 kJ/mol (Figure S1). As the GROMOS 45a3 parameter set was not parameterized to match experimental HFEs for polar compounds, such level of deviation was to be expected.

Due to a lack of pertinent experimental data, seven parameterized PTMs (carboxylysine, homocitrulline, citrulline, S-carbamoyl-cysteine, S-nitrosocysteine, 2-oxo-histidine and pyruvic acid) have remained unrepresented in the validation set, and therefore unverified in terms of reproducing experimental HFEs. To further assess the quality of the parameters for these compounds, we have compared them to those obtained by the Automated Topology Builder [26], a widely used online service for automated parameterization of small molecules compatible with the GROMOS 54a7 force field. While manually curated approaches are arguably superior to automated ones, it is reassuring to see that the two sets of parameters match closely. For example, we have observed close agreement between the sets of partial charges obtained using the two methods for these seven compounds, with a Pearson correlation coefficient *R* of 0.93 and an overall RMSD of 0.2 e^−^.

### Comparison of physico-chemical properties of PTMs and canonical amino acids

**Figure 3:**
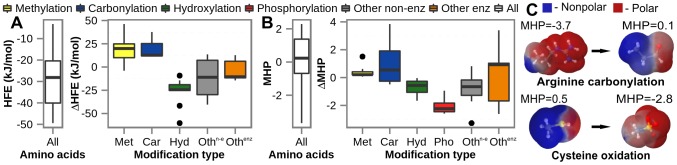
Hydrophobicity-related properties of PTMs compared to canonical amino acids. a) hydration free energies (HFEs) and b) molecular hydrophobicity potentials (MHPs). Distributions calculated of HFEs and MHPs of the canonical amino acids are captured using white boxes on the left side of both a) and b) panels. The distributions of HFE and MHP changes upon different types of PTMs are shown in colored boxes sorted according to the median of the underlying distributions. The distributions are shown using the box-and-whisker plotting method. Color code: methylation-yellow, carbonylation-blue, hydroxylation-green, phosphorylation-red, other enzymatic modifications-gray, other non-enzymatic modification-orange and all-white; c) change in surface MHP upon arginine carbonylation and cysteine oxidation, modifications with the most positive and the most negative MHP change, respectively. Note that we have not taken N-acetylglucosamine into account for the HFE and MHP analysis, since glycosylation modifications predominantly result in carbohydrate chains attached to target residues, while we provide parameters for this carbohydrate only as the first one in a typical chain.

As an application of the newly developed PTM parameters, we focus on the changes in several key physico-chemical properties of amino acids introduced by PTMs. Interestingly, the majority of post-translationally modified amino acids are larger in size than their native counterparts, with more than 85% of PTMs increasing the molecular weight and more than 80% of PTMs increasing the solvent accessible surface area (SASA) of the affected residues (Table S3) as calculated on energy-minimized (using the GROMOS 54a7 parameter set) configurations of PTMs and canonical amino acids. What is more, PTMs introduce significant changes in the electrostatic properties of target residues as illustrated in the case of net charge and dipole moment (Table S3). For example, 42% of all PTMs studied here undergo a charge change of 1 e^−^ or more in absolute value, with 88% of such changes resulting in a more negatively charged species. Moreover, the average absolute value of the change in dipole moment upon PTM equals 1.7 Debye, which is comparable in magnitude to the average dipole moment of 2.7 Debye or its standard deviation of 1.9 Debye as calculated in both cases over all unmodified residues using GROMOS 54a7 parameters and energy-minimized configurations. Finally, given the general importance of hydrophobicity in various biological processes, it is critical to understand in a quantitative manner how PTMs modulate the hydrophobicity of target amino acids. To address this question, we have used TI and GROMOS 54a7 parameters to calculate HFEs of all parameterized PTMs in neutral protonation states, since the available experimental data is insufficient for such an analysis. Our results show that methylation and carbonylation modifications increase HFEs on average by 18.6 kJ/mol and 20.5 kJ/mol, respectively, while hydroxylation modifications exhibit an opposite effect and decrease HFEs by on average 25.1 kJ/mol (Figure 3a). These changes are extremely relevant if one considers the fact that the two central quartiles of the distribution of HFEs for canonical amino acids span the range from approximately −40 kJ/mol to −20 kJ/mol (Figure 3a). Furthermore, the most extreme cases, i.e. symmetric di-methylation of arginine (ΔHFE = 46.2 kJ/mol) and di-hydroxylation of phenylalanine (ΔHFE = −60.3 kJ/mol) are comparable in absolute values to the total span of the canonical amino acid HFEs (−49.4 kJ/mol to −3.2 kJ/mol, Figure 3a). In other words, the effect of some PTMs on the HFEs of target amino acids is as large as the difference which would arise by mutating the most hydrophobic to the most hydrophilic canonical amino acid or vice versa. While some of these effects agree well with what one would qualitatively expect, for a number of PTMs our results are the first to provide a quantitative framework for such an analysis.

As both calculation and experimental measurement of HFEs are limited to neutral compounds only, the above analysis does not take into account charged modifications such as phosphorylation. To address this, we have used the molecular hydrophobicity potential (MHP) [27] approach to estimate hydrophobicity of all parameterized PTMs using their protonation states at physiological pH. MHP values are semi-empirical estimates of *logP*, a given compound's partition coefficient between water and the non-polar solvent octanol and are widely used in computational drug design [28], [29]. Similarly to the HFEs analysis, MHP calculations show that carbonylation and methylation are hydrophobicity-increasing modifications (Figure 3b), in contrast to phosphorylation and hydroxylation, which are hydrophilicity-increasing modifications. Finally, this analysis shows that PTMs can drastically change hydrophobic/hydrophilic properties of affected residues, e.g. arginine carbonylation shifts a highly hydrophilic to a highly hydrophobic residue, while cysteine oxidation does exactly the opposite (Figure 3c). By changing the chemical nature of affected residues, PTMs frequently completely alter their physico-chemical properties such as hydrophobicity, a feature with potentially far-reaching biological implications [11], [12], [30].

## Discussion

Despite the importance of understanding PTMs at the molecular level, MD simulations of post-translationally modified proteins lag significantly behind the studies of unmodified proteins, and this seems primarily due to a general lack of suitable computational tools and simulation parameters for treating PTMs. This study is to the best of our knowledge the first-ever effort to develop force-field parameters for the large majority of known PTMs in a systematic fashion. We have generated GROMOS force field (45a3 and 54a7) parameters for over 250 different enzymatic and non-enzymatic PTMs, spanning a wide range of modification types with a close to complete coverage of experimentally verified PTMs (Figure 1). Since GROMOS 54a7 force field parameters were fitted to reproduce experimental HFEs, we have tested the quality of the PTM parameters, obtained by manually curating the parameters of different groups mostly in analogy to canonical amino acids, by comparing the calculated HFEs against the experimental values. The newly generated parameters compatible with the GROMOS 54a7 parameter set reproduce experimental HFEs almost equally well as the original ones (Table 1 and Figure 2). Overall, only a few parameterized PTMs have not been directly validated against experimental HFEs due to a lack of experimentally available data. In those cases, however, good matching with the parameters obtained using an orthogonal, fully automated approach [26] lends support to the general validity of the reported parameters. However, one should emphasize that the full range of validity of the presented parameters could and should be delineated only by directly comparing MD simulations of different post-translationally modified proteins in biologically relevant contexts with relevant experimental data.

To date, PTMs in MD simulations have been treated in separate studies using different procedures and force fields, typically focusing on a single modification at a time [11], [13], [16]. Additionally, there are some available tools for automated generation of parameters (e.g. the AMBER [31] feature *antechamber* and online tools SwissParam [32], PRODRG [33], ATB [26] and *q4md-forcefieldtools* [34]), however, envisioned for small molecules rather than protein PTMs. The parameters reported herein have comparative advantage over these sources along three principal directions. First, we provide exclusively human curated and validated PTM force-field parameters, which are mutually fully consistent as well as being consistent with canonical amino acids. Second, we provide PTM parameters in both GROMOS [35] and GROMACS [36] format, widely used MD simulation packages (supporting GROMOS version 11 and GROMACS versions 3.× and newer), suitable for immediate simulation of modified proteins without any additional work required. This should be contrasted with the above tools that provide parameters for isolated compounds only. Finally, in combination with a publicly available online tool for introducing PTMs of choice to a user-supplied protein 3D structure (Vienna-PTM server, http://vienna-ptm.univie.ac.at) [37], we provide a comprehensive, user-friendly toolkit for studying PTMs using MD simulations.

During their lifecycle in the cell, almost all proteins undergo one or more different PTMs affecting their structure, dynamics and interaction networks and, subsequently, their function through direct alteration of chemical and physico-chemical properties of target residues (Figure 3). The force field parameters presented here, together with the Vienna-PTM webserver, provide a systematic framework required to study the effects of PTMs using MD simulations. As a first step in this direction, we have here compared the hydrophobicity-related variables (HFEs and MFP values) of native and modified amino acids and quantitatively showed that PTMs can have an extremely strong, biologically significant effect in this context. It has already been documented that some PTMs exert their biological effect through a general modification of the hydrophobicity of their targets. For example, lysine trimethylation is known to directly affect the binding of retinoic acid receptors, which regulate genes involved in growth, differentiation and apoptosis, to their partners via an increase in site-specific hydrophobicity [38]. Moreover, acetylated and methylated lysine residues in histones, i.e., some of the key components of the histone code, are recognized by the hydrophobic binding pockets of bromo- and chromo-domains based on the difference in hydrophobicity between the modified and unmodified lysines [39]. Furthermore, we have recently shown that carbonylation, which affects lysine, arginine, proline and threonine residues, drastically increases local propensity for aggregation in proteins by affecting the hydrophobicity of the modified sites [11]. While other, more specific effects of PTMs on the structure, dynamics and interaction profile of target proteins are certainly important, a major change in hydrophobicity, net charge, isoelectric point or any other general physico-chemical property caused by a PTM at a given site could certainly have major biological repercussions. We believe that our present study will provide a solid foundation for exploring such timely and important issues in the future. However, this is only one possible application of the PTM force-field parameters reported herein. From direct MD simulations to biomolecular structure refinement to computational free energy estimation and drug design, these parameters expand the range of MD methodLology to a large class of biomolecular systems of paramount importance. It is our hope that this advance will play a catalytic role in bringing together realistic cell biology, dominated by PTMs, and the quantitative, reductionist power of structural biology and chemistry, as embodied in the MD method, and help shed light on a broad spectrum of important biological questions at the microscopic level.

## Methods

### Parameterization of PTMs

One of the aims of the GROMOS force fields is to allow for the transfer of parameters between chemically similar groups in different compounds. Accordingly, we have derived GROMOS 45a3 and 54a7 force field parameters describing 110 post-translationally modified amino acids and protein termini (Table S1) by either novel parameterization or direct transfer from or analogy to already parameterized compounds including amino acids, nitrogenous bases and other small molecules according to the following principles and rationales.

General principles:

- Parameters were directly transferred from chemically identical groups (e.g. from the hydroxyl group of tyrosine to the hydroxyl group of 7-hydroxytryptophan) if such exist among parameterized compounds. If not, parameters were either directly transferred or inferred by analogy to the chemically most similar parameterized compound.
- Partial charges were assigned to add up to an integer net charge for every charge group, primarily by adjusting partial charges of less exposed atoms (e.g. the phosphorus atom of phospho-residues), while keeping them intact for terminal, more exposed atoms to affect interactions with other compounds as little as possible.

Modification type-specific principles:

1. PHOSPHORYLATION: Parameters directly transferred from phosphate and hydroxyl groups of nucleotides (e.g. ATP). The partial charge on the phosphorus atom fixed to get an integral net charge of a parameterized compound (dependent on the protonation state). The rest of a parameterized compound left unchanged. Additionally, analogy to the ester group reported by Chandrasekhar and others [40] used for phosphoaspartate.
2. METHYLATION: Parameters directly transferred or derived by analogy to different methyl-containing groups depending on the net charge and chemical context as follows:
  - directly transferred or derived by analogy from amines reported by Oostenbrink and others [41] for methylated lysine and histidine residues,
  - directly transferred or derived by analogy from nucleotides (e.g. ATP), arginine and amines reported by Oostenbrink and others [41] for methylated arginine residues,
  - derived by analogy to the peptide bond and the cognate native residues for methyl-asparagine and methyl-glutamine,
  - directly transferred from the ester group reported by Chandrasekhar and others [40] for aspartate methyl ester and glutamate methyl ester,
  - directly transferred from methionine for S-methylcysteine.
3. ACETYLATION: Parameters derived by analogy to the peptide bond and the carboxamide group (e.g. glutamine).
4. HYDROXYLATION: Parameters directly transferred from the hydroxyl group of threonine or tyrosine, if attached to an aliphatic or aromatic carbon atom, respectively.
5. CARBOXYLATION: Parameters directly transferred from the carboxyl group (e.g. glutamate).
6. SULFATION: Parameters derived by analogy to the phosphate group of nucleotides (e.g. adenosine).
7. DEHYDRATION: Parameters directly transferred from aliphatic carbon atoms using a bond type with a shorter equilibrium distance to mimic the properties of the double bond.
8. BROMIDATION: Parameters directly transferred from 8-bromo-guanosine triphosphate reported by Hritz and Oostenbrink [42].
9. S-NYTROSILATION: The oxygen atom parameters directly transferred for the carbonyl group (e.g. the peptide bond), with the nitrogen and sulfur atom partial charges fixed to add up to 0 net charge.
10. CITRULLINATION: Parameters derived by analogy to the peptide bond and the carboxamide group (e.g. glutamine).
11. ALLYSINE FORMATION: The oxygen atom parameters directly transferred for the carbonyl group (e.g. glutamine), with the carbon and hydrogen atom derived by analogy to the aldehyde group reported by Dolenc and others [43].
12. GLYCOSYLATION: Parameters directly transferred from the peptide bond and monosaccharide molecules (e.g. glucose).
13. CARBONYLATION: The oxygen atom parameters directly transferred for the carbonyl group (e.g. glutamine), with the carbon and hydrogen atom derived by analogy to the aldehyde group reported by Dolenc and others [43].
14. OXIDATION: Parameters directly transferred from different oxygen-containing groups depending on the net charge and chemical context:
  - from the carbonyl group (e.g. glutamine) and the phosphate group of nucleotides (e.g. adenosine) for methionine sulfoxide and methionine sulfone, respectively, with the partial charge of the sulphur atom fixed to get 0 net charge for oxidative modifications of methionine,
  - from the carboxyl group (e.g. glutamate) for cysteine oxidation modifications,
  - from the carbonyl group (e.g. glutamine) for the remaining oxidation modifications.
15. NITRATION: The oxygen atom parameters directly transferred from different oxygen-containing groups, with the nitrogen and carbon atoms partial charges adjusted to add up to an integer net charge, depending on the protonation state and chemical context; or derived *de novo* to match the experimental HFE of 2-nitrophenol:
  - from the base-linked oxygen atom of the phosphate group of nucleotides (e.g. adenosine) for the protonated forms of nitrotyrosine and nitrotryptophan,
  - derived *de novo* to match the HFE of 2-nitrophenol for the protonated form of nitrotyrosine,
  - from the base-linked oxygen atom of the phosphate group of nucleotides (e.g. adenosine) and the carboxyl group (e.g. glutamate) for the nitro and carboxyl groups, respectively, of the deprotonated form of nitrotyrosine.
16. KYNURENINE FORMATION: Parameters directly transferred from the carbonyl group (e.g. glutamine), the peptide bond and the amine group of the deprotonated arginine, with the carbon and hydrogen atom derived by analogy to the aldehyde group reported by Dolenc and others [43] for formyl-kynurenine.
17. CHLORINATION: Parameters directly transferred from chloroform.
18. CARBAMYLATION: Parameters directly transferred from the peptide bond, carboxyl group (e.g. glutamate) and the carboxamide group (e.g. glutamine).
19. NORLEUCINE: Parameters directly transferred from aliphatic carbon atoms.
20. N-TERMINAL METHYLATION: Parameters directly transferred from lysine methylation.
21. N-TERMINAL ACETYLATION: Parameters directly transferred from lysine acetylation.
22. N-TERMINAL PYRROLIDONE FORMATION: Parameters directly transferred from proline oxidation.
23. N-TERMINAL FORMYLATION: Parameters directly transferred from the peptide bond with the carbon and hydrogen atoms derived by analogy to the aldehyde group reported by Dolenc and others [43].
24. N-TERMINAL PYRUVATE FORMATION: Parameters directly transferred from the carbonyl group (e.g. glutamine), with a bond type of a shorter equilibrium distance used between the carbonyl carbon atoms to account for the double bond effect.
25. C-TERMINAL AMIDATION: Parameters directly transferred from the carboxamide group of e.g. glutamine.
26. C-TERMINAL METHYLATION: Parameters directly transferred from the ester group reported by Chandrasekhar and others [40].

We include detailed descriptions of parameter choices as comments in Dataset S1 and Dataset S2.

### Molecular dynamics simulations and thermodynamic integration setup

We have used the thermodynamic integration approach [25], a widely used computational method based on MD simulations, to calculate hydration free energies (HFEs) of neutral forms of small-molecule analogs of 14 amino-acid side chains (the same set as in Oostenbrink et al. [21]), compounds from the validation set and side chain analogs of all parameterized PTMs with a charge neutral protonation state. Non-bonded (van der Waals and Coulomb) interactions, coupled to a parameter λ, were scaled down to zero in a stepwise manner in vacuum and water. Free energy changes of these processes were calculated as the integral of the ensemble average of the derivative of the total Hamiltonian of the system with respect to λ, over the interval from λ = 0 to λ = 1. For vacuum calculations, three independent simulations, each 5 ns long, were run at 21 equally spaced λ-points with the temperature kept at 500 K and additional random kicks introduced by Langevin dynamics integration method [44], in order to avoid convergence problems due to inefficient sampling of the conformational space. Water simulations were run in five independent copies, each 0.5 ns long, at 21 equally spaced λ-points, together with 10 additional λ-points placed in the regions of the least smoothness of the integrated curve, using SPC explicit water [45], a reaction field electrostatic scheme with a cutoff of r_c_ = 1.4 nm and the dielectric constant of ε_rf_ = 65 and a Berendsen thermostat and barostat keeping the temperature and pressure at 300 K (τ*_T_* = 0.05 ps) and 1 bar (τ*_p_* = 1 ps and compressibility = 4.5×10^−5^ bar^−1^) [46]. A soft-core formalism [47] was used to avoid singularities of the potential energy. The aforementioned integrals were evaluated by the generalized Simpson's rule for non-equidistant nodes using the averages over the independent simulations at each λ-point. HFEs were calculated as the difference between the change in free energy upon the removal of non-bonded interactions calculated in vacuum and calculated in water.

## Supporting Information

Dataset S1 **Force field parameters for the GROMOS force field 45A3 parameter set.**
(TXT)

Dataset S2 **Force field parameters for the GROMOS force field 54A7 parameter set.**
(TXT)

Dataset S3 **UniProt entries of post-translationally modified proteins.**
(TXT)

Figure S1 **Experimental vs. calculated HFEs of compounds from the validation set (GROMOS 45a3).** Correlation is captured by the regression line, its parameters, Pearson correlation coefficient and overall RMSE. The same comparison for canonical amino acids is shown in the inset. Note that error bars of calculated HFEs are comparable to the size of the symbols, with the average standard error of 0.4 kJ/mol.
(TIF)

Table S1 **Parameterized post-translational modifications with the 3-letter code, chemical names and structures.** If two protonation states are possible, the one with higher occupancy at the physiologic pH is highlighted in bold. Note that modifications marked with: 1) * were already parameterized in GROMOS force field, 2) **^#^** have to date not been reported in UNIPROT, 3) **^+^** no prolines included and 4) ^HFE^ parameters derived to match the experimental HFE.
(PDF)

Table S2 **HFEs of the molecules in the validation set, comparison between the experimental and calculated values using the GROMOS 45a3 parameter set.**
(PDF)

Table S3 **Comparison of physico-chemical properties of PTMs and canonical amino acids.** Molecular weight (MW), solvent accessible surface area (SASA), net charge and dipole moment (DM) shown for PTMs and cognate amino acids in parentheses.
(PDF)
