# Supplementary figures and images for: A Systematic Framework for Molecular Dynamics Simulations of Protein Post-Translational Modifications
Source: PLoS Comput Biol. 2013 Jul 18;9(7):e1003154. doi: 10.1371/journal.pcbi.1003154 (PMC3715417; doi:10.1371/journal.pcbi.1003154)

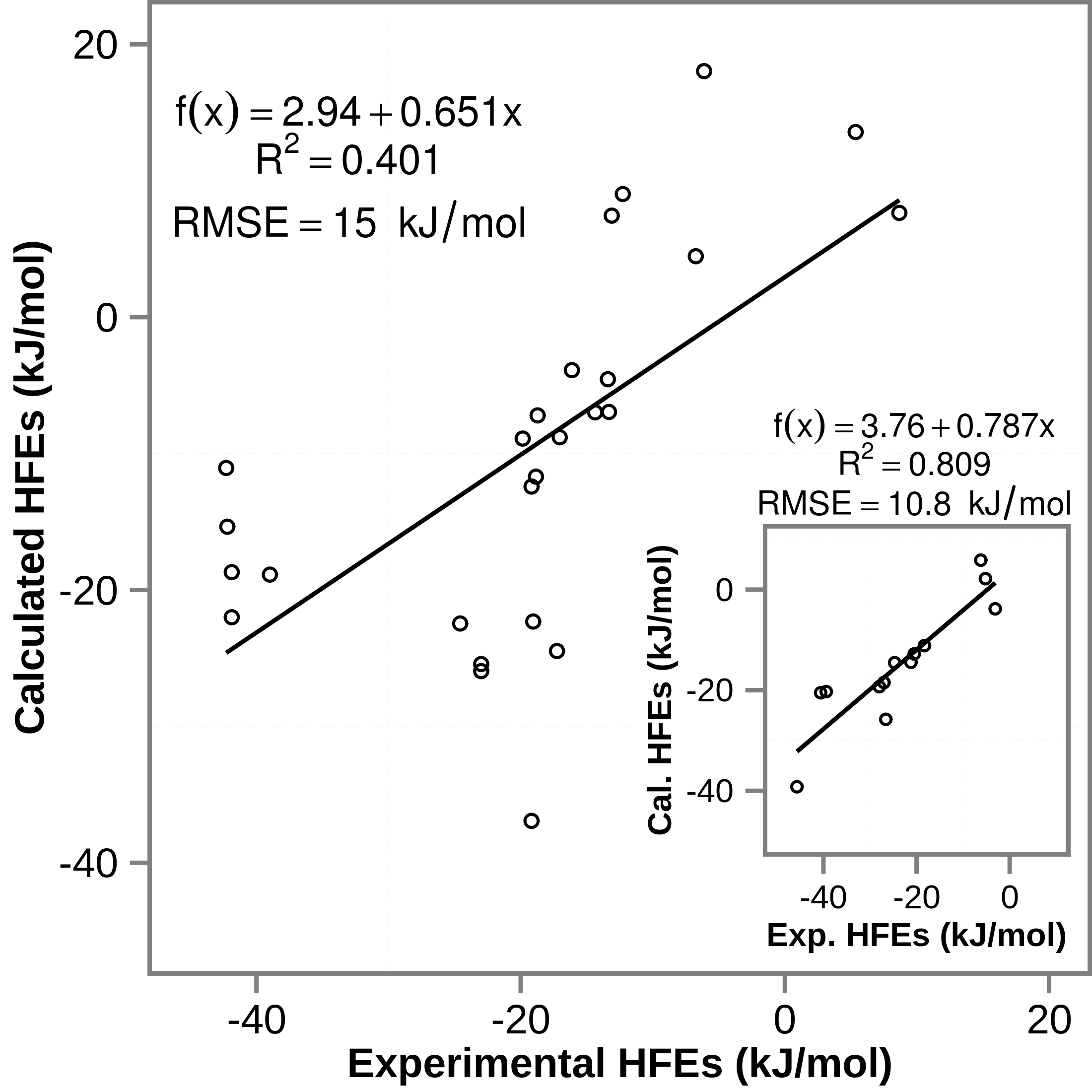

Supplement: Figure S1 — Experimental vs. calculated HFEs of compounds from the validation set (GROMOS 45a3). Correlation is captured by the regression line, its parameters, Pearson correlation coefficient and overall RMSE. The same comparison for canonical amino acids is shown in the inset. Note that error bars of calculated HFEs are comparable to the size of the symbols, with the average standard error of 0.4 kJ/mol. (TIF) [file pcbi.1003154.s004.tif]
