# Supplementary material for: A Systematic Framework for Molecular Dynamics Simulations of Protein Post-Translational Modifications
Source: PLoS Comput Biol. 2013 Jul 18;9(7):e1003154. doi: 10.1371/journal.pcbi.1003154 (PMC3715417; doi:10.1371/journal.pcbi.1003154)
Supplement: Table S1 — Parameterized post-translational modifications with the 3-letter code, chemical names and structures. If two protonation states are possible, the one with higher occupancy at the physiologic pH is highlighted in bold. Note that modifications marked with: 1) * were already parameterized in GROMOS force field, 2) # have to date not been reported in UNIPROT, 3) + no prolines included and 4) HFE parameters derived to match the experimental HFE. (PDF) [file pcbi.1003154.s005.pdf]

ENZYMATIC

Phosphorylation

# PTM		AA	code	chemical	structure
1	1	SER	S1P	phosphoserine (-1)	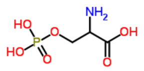
2	2		**S2P**	phosphoserine (-2)	
3	3	THR	T1P	phosphothreonine (-1)	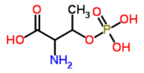
4	4		**T2P**	phosphothreonine (-2)	
5	5	TYR	Y1P	phosphotyrosine (-1)	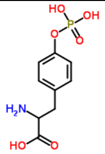
6	6		**Y2P**	phosphotyrosine (-2)	
7	7	ASP	D1P	phosphoaspartate (-1)	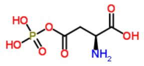
8	8		**D2P**	phosphoaspartate (-2)	
9	9	LYS	K1P[#]	phospholysine (-1)	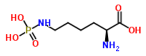
10	10		**K2P**[#]	phospholysine (-2)	
11	11	ARG	R0P[#]	phosphoarginine (0)	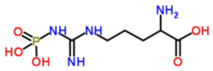
12	12		**R1P**[#]	phosphoarginine (-1)	
13	13	HIS	H11	1-phosphohistidine (-1)	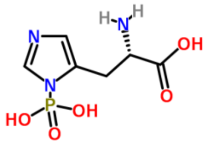
14	14		**H12**	1-phosphohistidine (-2)	
15	15	HIS	H31	3-phosphohistidine (-1)	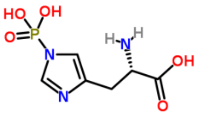
16	16		**H32**	3-phosphohistidine (-2)	

Methylation

17	17	LYS	KMN	N6-methyllysine (0)	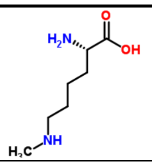
18	18		**KMC**	N6-methyllysine (+1)	

19	19	LYS	K2M	N6,N6-dimethyllysine (0)	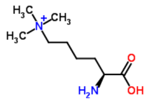
20	20		**K2C**	N6,N6-dimethyllysine (+1)	
21	21	LYS	K3C	N6,N6,N6-trimethyllysine	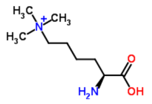
22	22	ARG	RMN	omega-N-methylarginine (0)	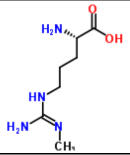
23	23		**RMC**	omega-N-methylarginine (+1)	
24	24	ARG	RSM	symmetric-dimethylarginine (0)	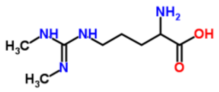
25	25		**RMS**	symmetric-dimethylarginine (+1)	
26	26	ARG	RAM	asymmetric-dimethylarginine (0)	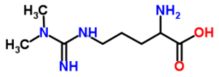
27	27		**RMA**	asymmetric-dimethylarginine (+1)	
28	28	HIS	**H1M**	1-methylhistidine (0)	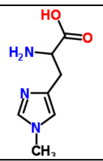
29	29		H1C	1-methylhistidine (+1)	
30	30	HIS	**H3M**	3-methylhistidine (0)	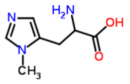
31	31		H3C	3-methylhistidine (+1)	
32	32	GLN	QME	N5-methylglutamine	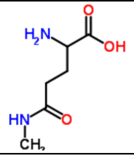
33	33	ASN	NME	N4-methylasparagine	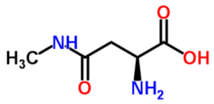
34	34	GLU	EME	glutamate methyl ester	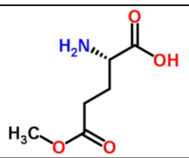
35	35	ASP	DMA[#]	aspartate methyl ester	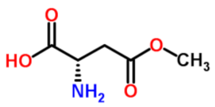
36	36	CYS	CYM	S-methylcysteine	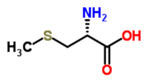

Acetylation

37	37	LYS	KAC	N6-acetyllysine	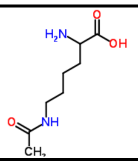

Hydroxylation

38	38	PRO	PH3	3-hydroxyproline (R)	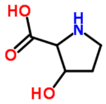
39	39		P3H	3-hydroxyproline (S)	
40	**1***	PRO	HYP	4-hydroxyproline (R)	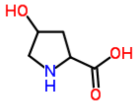
41	40		HY2	4-hydroxyproline (S)	
42	41	PRO	PHH	3,4-dihydroxyproline	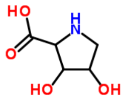
43	42	LYS	KH5	5-hydroxylysine (0,R)	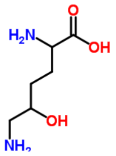
44	43		K5H	5-hydroxylysine (0,S)	
45	44		**KPH**	5-hydroxylysine (+1,R)	
46	45		**KHP**	5-hydroxylysine (+1,S)	
47	46	TYR	HTY	3,4-dihydroxyphenylalanine	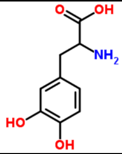
48	47	TRP	W7H	7-hydroxytryptophan	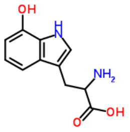
49	48	ASP	**DH3**	3-hydroxyaspartate (-1,R)	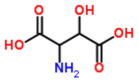
50	49		**D3H**	3-hydroxyaspartate (-1,S)	
51	50		DN3	3-hydroxyaspartate (0,R)	
52	51		D3N	3-hydroxyaspartate (0,S)	
53	52	ASN	N3H	3-hydroxyasparagine (R)	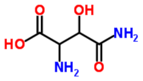
54	53		NH3	3-hydroxyasparagine (S)	

Carboxylation

55	54	GLU	**ECA**	4-carboxyglutamate (-2)	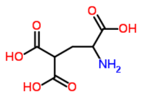
56	55		ECN	4-carboxyglutamate (-1)	

Sulfation

57	56	TYR	YSU	sulfotyrosine	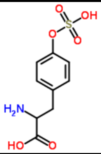

Dehydration

58	57	SER	SDH	dehydroalanine	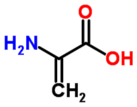
59	58	THR	TDH	2,3-didehydrobutyrine	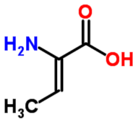

Bromidation

60	59	TRP	WBR	6-bromotryptophan	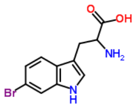

S-nitrosylation

61	60	CYS	CSN	S-nitrosocysteine	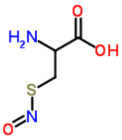

Citrullination

62	61	ARG	RCI	citrulline	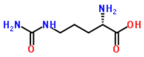

Allysine formation (the same as carbonylation)

63	62	LYS	KAL	allysine (aminoadipic semialdehyde)	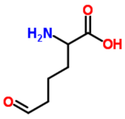

Glycosylation

64	63	ASN	NNG	N-acetylglucosamine (N4-linked to ASN)	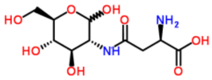

NONENZYMATIC

# PTM		AA	Code	chemical	structure
**Hydroxylation**					
65	64	PHE	F23#	2,3-dihydroxyphenylalanine	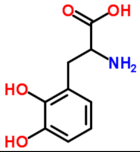
66	65	PHE	F2H#	2-hydroxyphenylalanine	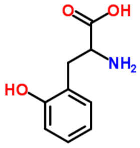
67	66	PHE	F3H#	3-hydroxyphenylalanine	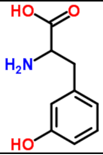
68	**2***	PHE	TYR#	tyrosine	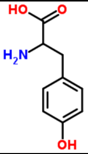
69	67	TRP	W6H#	6-hydroxytryptophan	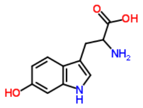
70	68	TRP	W5H#	5-hydroxytryptophan	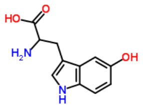
71	69	TRP	W4H#	4-hydroxytryptophan	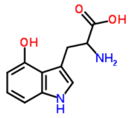
72	70	TRP	W2H#	2-hydroxytryptophan	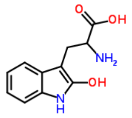
73	71	LEU	L3H#	3-hydroxyleucine (R)	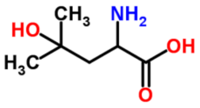
74	72		LH3#	3-hydroxyleucine (S)	
75	73	LEU	L4H#	4-hydroxyleucine	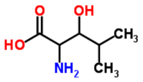

76	74	LEU	L5H#	5-hydroxyleucine (R)	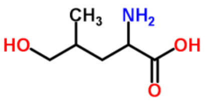
77	75		LH5#	5-hydroxyleucine (S)	
78	76	VAL	V3H	3-hyroxyvaline	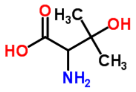
79	77	CYS	CYH	cysteine sulfenic acid	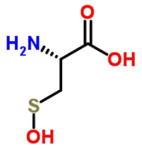
80	78	PRO	PH5#	5-hydroxyproline (R)	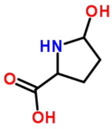
81	79		P5H#	5-hydroxyproline (S)	

Carbonylation

**63**	**62**	LYS	KAL	allysine (aminoadipic semialdehyde)	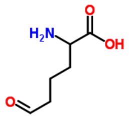
82	80	PRO	GSA#	glutamic semialdehyde	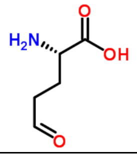
83		ARG			
84	81	THR	TOX#	2-amino-3-ketobutyric acid	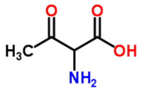

Oxidation

85	82	PRO	PGA#	pyroglutamic acid	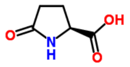
86	**3***	HIS	ASN	asparagine	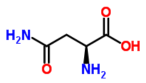
87	**4***	HIS	**ASP**	aspartic acid (-1)	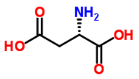
88	**5***		ASPH	aspartic acid (0)	
89	83	HIS	H2X#	2-oxo-histidine	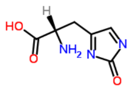

90	84	MET	MSX	methionine sulfoxide (R)	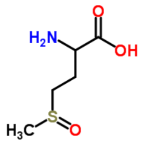
91	85		MXS	methionine sulfoxide (S)	
92	86	MET	MES	methionine sulfone	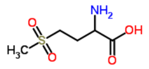
93	87	CYS	CSA	cysteine sulfinic acid	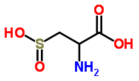
94	88	CYS	CSE[#]	cysteic acid	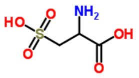

Nitration

95	89	TYR	**YNI**	3-nitrotyrosine (-1)	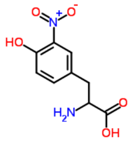
96	90		YNN[HFE]	3-nitrotyrosine (0)	
			YNB	3-nitrotyrosine (0)	
97	91	TRP	WNI[#]	6-nitrotryptophan	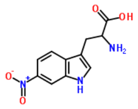

Kynurenine formation

98	92	TRP	WKY[#]	kynurenine	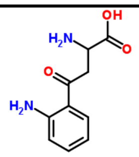
99	93	TRP	WKH[#]	3-hydroxykynurenine	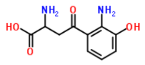
100	94	TRP	WKF[#]	formylkynurenine	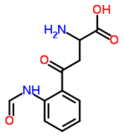

Chlorination

101	95	TYR	YCH[#]	chlorotyrosine	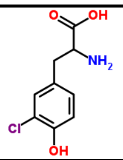

Deamidation

102	**4***	ASN	**ASP**	aspartic acid (-1)	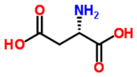
103	**5***		ASPH	aspartic acid (0)	
104	**6***	GLN	**GLU**	glutamic acid (-1)	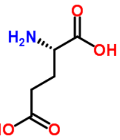
105	**7***		GLUH	glutamic acid (0)	

Carbamylation

106	96	LYS	KAM[#]	homocitruline	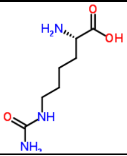
107	97	LYS	**KCA**	carboxylysine (+1)	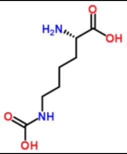
108	98		KCN	carboxylysine (0)	
109	99	CYS	CAM[#]	S-carbamoylcysteine	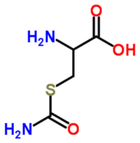

Norleucine

110	100	LEU	LNO[#]	norleucine	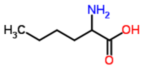
111		LYS			
112		MET			

N-TERMINAL

# PTM		AA	Code	chemical	structure

Methylation

113-132	101	all	1NM	N-methyl-AA (0)	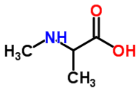
133-152	102		**1NM+**	N-methyl-AA (+1)	

153-171	103	**all**$^{+}$	2NM	N,N-dimethyl-AA (0)	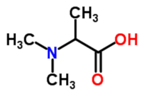
172-191	104		**2NM+**	N,N-dimethyl-AA (+1)	
193-210	105	**all**$^{+}$	3NM+	N,N,N-trimethyl-AA	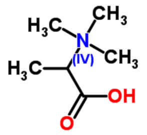

Acetylation

211-230	106	all	NAC	N-acetyl-AA	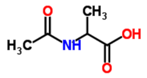

Pyrrolidone formation

231	**81**	**GLN**	PGA	pyroglutamic acid	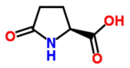
232		GLU			

Formylation

233	107	MET	FOR	N-formylmethionine	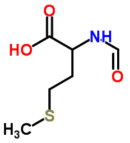

Pyruvate formation

234	108	**SER**	PYA	pyruvic acid	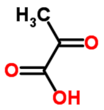
235		CYS			
236		VAL			

C-TERMINAL

# PTM		AA	Code	chemical	structure

Amidation

237-256	109	all	AMD	AA-amide	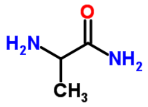

Methylation

257	110	**CYS**	CME	AA-methyl ester	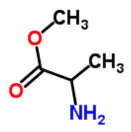
258		LEU			
259		LYS			
