# Supplementary material for: A Systematic Framework for Molecular Dynamics Simulations of Protein Post-Translational Modifications
Source: PLoS Comput Biol. 2013 Jul 18;9(7):e1003154. doi: 10.1371/journal.pcbi.1003154 (PMC3715417; doi:10.1371/journal.pcbi.1003154)
Supplement: Table S2 — HFEs of the molecules in the validation set, comparison between the experimental and calculated values using the GROMOS 45a3 parameter set. (PDF) [file pcbi.1003154.s006.pdf]

Compound	HFE (kJ/mol)	
	**experimental**	**ffG45a3**
**Validation set. PTM-side-chain analogs**		
N-butylacetamide	-39.0	-18.9
o-cresol	-24.6	-22.5
m-cresol	-23.0	-25.9
2-methyl-2-propanol	-18.7	-7.2
2-methyl-1-propanol	-18.8	-11.7
propan-2-ol	-19.8	-8.9
N-methylacetamine	-41.9	-18.7
methylpropanoate	-12.3	9.0
methylacetate	-13.1	7.4
dimethylsulfide	-6.7	4.5
butanal	-13.3	-7.0
propanal	-14.4	-7.0
butane	8.7	7.6
**Validation set. Compounds similar to PTM-side-chain analogs**		
diethylamine	-17.0	-8.8
trimethylamine	-13.4	-4.6
ethene	5.4	13.6
bromobenzene	-6.1	18.0
aniline	-23.0	-25.4
acetophenone	-19.2	-12.4
N-methylformamide	-41.9	-22.0
chlorophenol	-19.0	-22.3
2-nitrophenol	-19.2	-36.9
nitrobenzene	-17.2	-24.5
acetone	-16.1	-3.9
dimethylsulfoxide	-42.3	-11.1
methylsulfonylmethane	-42.2	-15.4
**RMSE**	**-**	**15.0**
