# Supplementary material for: A Systematic Framework for Molecular Dynamics Simulations of Protein Post-Translational Modifications
Source: PLoS Comput Biol. 2013 Jul 18;9(7):e1003154. doi: 10.1371/journal.pcbi.1003154 (PMC3715417; doi:10.1371/journal.pcbi.1003154)
Supplement: Table S3 — Comparison of physico-chemical properties of PTMs and canonical amino acids. Molecular weight (MW), solvent accessible surface area (SASA), net charge and dipole moment (DM) shown for PTMs and cognate amino acids in parentheses. (PDF) [file pcbi.1003154.s007.pdf]

PTM (AA)	chemical	MW (u)	SASA (nm^2)	net charge	DM (D)
S1P (SER)	phosphoserine (-1)	110.0 (31.0)	1.88 (1.20)	-1 (0)	N/A (2.30)
S2P (SER)	phosphoserine (-2)	109.0 (31.0)	1.93 (1.20)	-2 (0)	N/A (2.30)
T1P (THR)	phosphothreonine (-1)	124.0 (45.1)	2.12 (1.41)	-1 (0)	N/A (2.23)
T2P (THR)	phosphothreonine (-2)	123.0 (45.1)	2.15 (1.41)	-2 (0)	N/A (2.23)
Y1P (TYR)	phosphotyrosine (-1)	186.1 (107.1)	2.81 (2.41)	-1 (0)	N/A (2.06)
Y2P (TYR)	phosphotyrosine (-2)	185.1 (107.1)	2.73 (2.41)	-2 (0)	N/A (2.06)
D1P (ASP)	phosphoaspartate (-1)	138.0 (58.0)	2.23 (1.57)	-1 (-1)	N/A (N/A)
D2P (ASP)	phosphoaspartate (-2)	137.0 (58.0)	2.17 (1.57)	-2 (-1)	N/A (N/A)
K1P (LYS)	phospholysine (-1)	151.1 (73.1)	2.59 (1.92)	-1 (1)	N/A (N/A)
K2P (LYS)	phospholysine (-2)	150.1 (73.1)	2.54 (1.92)	-2 (1)	N/A (N/A)
R0P (ARG)	phosphoarginine (0)	180.1 (101.2)	2.82 (2.21)	0 (1)	14.65 (N/A)
R1P (ARG)	phosphoarginine (-1)	179.1 (101.2)	2.73 (2.21)	-1 (1)	N/A (N/A)
H11 (HIS)	1-phosphohistidine (-1)	160.1 (81.1)	2.36 (1.91)	-1 (0)	N/A (6.43)
H12 (HIS)	1-phosphohistidine (-2)	159.1 (81.1)	2.39 (1.91)	-2 (0)	N/A (6.43)
H31 (HIS)	3-phosphohistidine (-1)	160.1 (81.1)	2.51 (1.91)	-1 (0)	N/A (6.43)
H32 (HIS)	3-phosphohistidine (-2)	159.1 (81.1)	2.53 (1.91)	-2 (0)	N/A (6.43)
KMN (LYS)	N6-methyllysine (0)	86.2 (73.1)	2.40 (1.92)	0 (1)	1.84 (N/A)
KMC (LYS)	N6-methyllysine (+1)	87.2 (73.1)	2.26 (1.92)	1 (1)	N/A (N/A)
K2M (LYS)	N6,N6-dimethyllysine (0)	100.2 (73.1)	2.72 (1.92)	0 (1)	1.58 (N/A)
K2C (LYS)	N6,N6-dimethyllysine (+1)	101.2 (73.1)	2.45 (1.92)	1 (1)	N/A (N/A)
K3C (LYS)	N6,N6,N6-trimethyllysine	115.2 (73.1)	2.68 (1.92)	1 (1)	N/A (N/A)
RMN (ARG)	omega-N-methylarginine (0)	114.2 (101.2)	2.38 (2.21)	0 (1)	2.78 (N/A)
RMC (ARG)	omega-N-methylarginine (+1)	115.2 (101.2)	2.43 (2.21)	1 (1)	N/A (N/A)
RSM (ARG)	symmetric-dimethylarginine (0)	128.2 (101.2)	2.80 (2.21)	0 (1)	0.94 (N/A)
RMS (ARG)	symmetric-dimethylarginine (+1)	129.2 (101.2)	2.69 (2.21)	1 (1)	N/A (N/A)

RAM (ARG)	asymmetric-dimethylarginine (0)	128.2 (101.2)	2.62 (2.21)	0 (1)	3.55 (N/A)
RMA (ARG)	asymmetric-dimethylarginine (+1)	129.2 (101.2)	2.69 (2.21)	1 (1)	N/A (N/A)
H1M (HIS)	1-methylhistidine (0)	95.1 (81.1)	2.24 (1.91)	0 (0)	6.10 (6.43)
H1C (HIS)	1-methylhistidine (+1)	96.1 (81.1)	2.19 (1.91)	1 (0)	N/A (6.43)
H3M (HIS)	3-methylhistidine (0)	95.1 (81.1)	2.32 (1.91)	0 (0)	4.33 (6.43)
H3C (HIS)	3-methylhistidine (+1)	96.1 (81.1)	2.01 (1.91)	1 (0)	N/A (6.43)
QME (GLN)	N5-methylglutamine	86.1 (72.1)	2.07 (1.80)	0 (0)	2.28 (4.94)
NME (ASN)	N4-methylasparagine	72.1 (58.1)	1.97 (1.56)	0 (0)	4.14 (4.90)
EME (GLU)	glutamate methyl ester	87.1 (72.1)	1.99 (1.79)	0 (-1)	5.27 (N/A)
DMA (ASP)	aspartate methyl ester	73.1 (58.0)	1.80 (1.57)	0 (-1)	5.31 (N/A)
CYM (CYS)	S-methylcysteine	61.1 (47.1)	1.65 (1.41)	0 (0)	2.74 (1.84)
KAC (LYS)	N6-acetyllysine	114.2 (73.1)	2.67 (1.92)	0 (1)	4.12 (N/A)
PH3 (PRO)	3-hydroxyproline (R)	58.1 (42.1)	1.61 (1.59)	0 (0)	2.32 (N/A)
P3H (PRO)	3-hydroxyproline (S)	58.1 (42.1)	1.64 (1.59)	0 (0)	2.27 (N/A)
HYP (PRO)	4-hydroxyproline (R)	58.1 (42.1)	1.77 (1.59)	0 (0)	2.14 (N/A)
HY2 (PRO)	4-hydroxyproline (S)	58.1 (42.1)	1.67 (1.59)	0 (0)	2.20 (N/A)
PHH (PRO)	3,4-dihydroxyproline	74.1 (42.1)	1.76 (1.59)	0 (0)	3.66 (N/A)
KH5 (LYS)	5-hydroxylysine (0,R)	88.1 (73.1)	2.03 (1.92)	0 (1)	3.62 (N/A)
K5H (LYS)	5-hydroxylysine (0,S)	88.1 (73.1)	2.17 (1.92)	0 (1)	5.86 (N/A)
KPH (LYS)	5-hydroxylysine (+1,R)	89.1 (73.1)	2.05 (1.92)	1 (1)	N/A (N/A)
KHP (LYS)	5-hydroxylysine (+1,S)	89.1 (73.1)	2.03 (1.92)	1 (1)	N/A (N/A)
HTY (TYR)	3,4-dihydroxyphenylalanine	123.1 (107.1)	2.29 (2.41)	0 (0)	3.97 (2.06)
W7H (TRP)	7-hydroxytryptophan	146.2 (130.2)	2.70 (2.63)	0 (0)	3.19 (3.65)
DH3 (ASP)	3-hydroxyaspartate (-1,R)	74.0 (58.0)	1.52 (1.57)	-1 (-1)	N/A (N/A)
D3H (ASP)	3-hydroxyaspartate (-1,S)	74.0 (58.0)	1.57 (1.57)	-1 (-1)	N/A (N/A)
DN3 (ASP)	3-hydroxyaspartate (0,R)	75.0 (58.0)	1.61 (1.57)	0 (-1)	0.58 (N/A)

D3N (ASP)	3-hydroxyaspartate (0,S)	75.0 (58.0)	1.56 (1.57)	0 (-1)	3.79 (N/A)
N3H (ASN)	3-hydroxyasparagine (R)	74.1 (58.1)	1.71 (1.56)	0 (0)	3.34 (4.90)
NH3 (ASN)	3-hydroxyasparagine (S)	74.1 (58.1)	1.84 (1.56)	0 (0)	6.30 (4.90)
ECA (GLU)	4-carboxyglutamate (-2)	115.1 (72.1)	2.06 (1.79)	-2 (-1)	N/A (N/A)
ECN (GLU)	4-carboxyglutamate (-1)	116.1 (72.1)	2.20 (1.79)	-1 (-1)	N/A (N/A)
YSU (TYR)	sulfotyrosine	186.2 (107.1)	2.66 (2.41)	-1 (0)	N/A (2.06)
SDH (SER)	dehydroalanine	14.0 (31.0)	1.06 (1.20)	0 (0)	0.00 (2.30)
TDH (THR)	2,3-didehydrobutyrine	28.1 (45.1)	1.39 (1.41)	0 (0)	0.00 (2.23)
WBR (TRP)	6-bromotryptophan	209.1 (130.2)	2.88 (2.63)	0 (0)	3.16 (3.65)
CSN (CYS)	S-nitrosocysteine	76.1 (47.1)	1.71 (1.41)	0 (0)	3.15 (1.84)
RCI (ARG)	citrulline	101.1 (101.2)	2.22 (2.21)	0 (1)	5.21 (N/A)
KAL (LYS)	allysine (aminoadipic semialdehyde)	71.1 (73.1)	2.04 (1.92)	0 (1)	2.96 (N/A)
NNG (ASN)	N-acetylglucosamine	261.3 (58.1)	3.73 (1.56)	0 (0)	9.10 (4.90)
F23 (PHE)	2,3-dihydroxyphenylalanine	123.1 (91.1)	2.29 (2.28)	0 (0)	4.55 (0.74)
F2H (PHE)	2-hydroxyphenylalanine	107.1 (91.1)	2.13 (2.28)	0 (0)	3.11 (0.74)
F3H (PHE)	3-hydroxyphenylalanine	107.1 (91.1)	2.08 (2.28)	0 (0)	2.73 (0.74)
TYR (PHE)	tyrosine	107.1 (91.1)	2.41 (2.28)	0 (0)	2.06 (0.74)
W6H (TRP)	6-hydroxytryptophan	146.2 (130.2)	2.75 (2.63)	0 (0)	1.32 (3.65)
W5H (TRP)	5-hydroxytryptophan	146.2 (130.2)	2.71 (2.63)	0 (0)	3.46 (3.65)
W4H (TRP)	4-hydroxytryptophan	146.2 (130.2)	2.71 (2.63)	0 (0)	4.88 (3.65)
W2H (TRP)	2-hydroxytryptophan	146.2 (130.2)	2.73 (2.63)	0 (0)	5.19 (3.65)
L3H (LEU)	3-hydroxyleucine (R)	73.1 (57.1)	1.96 (1.88)	0 (0)	2.23 (0.00)
LH3 (LEU)	3-hydroxyleucine (S)	73.1 (57.1)	1.86 (1.88)	0 (0)	2.25 (0.00)
L4H (LEU)	4-hydroxyleucine	73.1 (57.1)	1.92 (1.88)	0 (0)	2.19 (0.00)
L5H (LEU)	5-hydroxyleucine (R)	73.1 (57.1)	1.98 (1.88)	0 (0)	2.17 (0.00)
LH5 (LEU)	5-hydroxyleucine (S)	73.1 (57.1)	2.01 (1.88)	0 (0)	2.19 (0.00)

V3H (VAL)	3-hyroxyvaline	59.1 (43.1)	1.70 (1.55)	0 (0)	2.30 (0.00)
CYH (CYS)	cysteine sulfenic acid	63.1 (47.1)	1.62 (1.41)	0 (0)	2.38 (1.84)
PH5 (PRO)	5-hydroxyproline (R)	58.1 (42.1)	1.69 (1.59)	0 (0)	2.21 (N/A)
P5H (PRO)	5-hydroxyproline (S)	58.1 (42.1)	1.79 (1.59)	0 (0)	2.17 (N/A)
GSA (PRO)	glutamic semialdehyde	57.1 (42.1)	1.64 (1.59)	0 (0)	2.95 (N/A)
GSA (ARG)	glutamic semialdehyde	57.1 (101.2)	1.64 (2.21)	0 (1)	2.95 (N/A)
TOX (THR)	2-amino-3-ketobutyric acid	43.0 (45.1)	1.40 (1.41)	0 (0)	2.66 (2.23)
PGA (PRO)	pyroglutamic acid	56.1 (42.1)	1.74 (1.59)	0 (0)	2.65 (N/A)
ASN (HIS)	asparagine	58.1 (81.1)	1.56 (1.91)	0 (0)	4.90 (6.43)
ASP (HIS)	aspartic acid (-1)	58.0 (81.1)	1.57 (1.91)	-1 (0)	N/A (6.43)
H2X (HIS)	2-oxo-histidine	95.1 (81.1)	2.00 (1.91)	0 (0)	9.64 (6.43)
MSX (MET)	methionine sulfoxide (R)	91.2 (75.2)	2.02 (1.94)	0 (0)	4.27 (2.62)
MXS (MET)	methionine sulfoxide (S)	91.2 (75.2)	2.11 (1.94)	0 (0)	4.23 (2.62)
MES (MET)	methionine sulfone	107.2 (75.2)	2.14 (1.94)	0 (0)	6.80 (2.62)
CSA (CYS)	cysteine sulfinic acid	78.1 (47.1)	1.69 (1.41)	-1 (0)	N/A (1.84)
CSE (CYS)	cysteic acid	94.1 (47.1)	1.80 (1.41)	-1 (0)	N/A (1.84)
YNI (TYR)	3-nitrotyrosine (-1)	151.1 (107.1)	2.61 (2.41)	-1 (0)	N/A (2.06)
YNN (TYR)	3-nitrotyrosine (0)	152.1 (107.1)	2.62 (2.41)	0 (0)	5.44 (2.06)
YNB (TYR)	3-nitrotyrosine (0)	152.1 (107.1)	2.62 (2.41)	0 (0)	3.83 (2.06)
WNI (TRP)	6-nitrotryptophan	175.2 (130.2)	3.06 (2.63)	0 (0)	3.08 (3.65)
WKY (TRP)	kynurenine	134.2 (130.2)	2.60 (2.63)	0 (0)	4.16 (3.65)
WKH (TRP)	3-hydroxykynurenine	150.2 (130.2)	2.66 (2.63)	0 (0)	2.78 (3.65)
WKF (TRP)	formylkynurenine	162.2 (130.2)	2.98 (2.63)	0 (0)	3.03 (3.65)
YCH (TYR)	chlorotyrosine	141.6 (107.1)	2.44 (2.41)	0 (0)	3.43 (2.06)
ASP (ASN)	aspartic acid (-1)	58.0 (58.1)	1.57 (1.56)	-1 (0)	N/A (4.90)
GLU (GLN)	glutamic acid (-1)	72.1 (72.1)	1.79 (1.80)	-1 (0)	N/A (4.94)

KAM (LYS)	homocitruline	115.2 (73.1)	2.46 (1.92)	0 (1)	5.24 (N/A)
KCA (LYS)	carboxylysine (+1)	115.1 (73.1)	2.48 (1.92)	-1 (1)	N/A (N/A)
KCN (LYS)	carboxylysine (0)	116.1 (73.1)	2.49 (1.92)	0 (1)	3.63 (N/A)
CAM (CYS)	S-carbamoylcysteine	90.1 (47.1)	1.99 (1.41)	0 (0)	4.81 (1.84)
LNO (LEU)	norleucine	57.1 (57.1)	1.88 (1.88)	0 (0)	0.00 (0.00)
LNO (LYS)	norleucine	57.1 (73.1)	1.88 (1.92)	0 (1)	0.00 (N/A)
LNO (MET)	norleucine	57.1 (75.2)	1.88 (1.94)	0 (0)	0.00 (2.62)
